# The Menstrual Cycle and Risk of Breast Cancer: A Review

**DOI:** 10.3389/fonc.2020.00021

**Published:** 2020-01-24

**Authors:** Håkan Lars Olsson, Mona Landin Olsson

**Affiliations:** ^1^Departments of Oncology and Cancer Epidemiology, Clinical Sciences, Lund University, Lund, Sweden; ^2^Department of Endocrinology, Clinical Sciences, Lund University, Lund, Sweden

**Keywords:** breast cancer, menstrual cycle, risk, retrospective, prospective

## Abstract

Cyclic hormonal stimulation of the breast tissue plays a significant role in breast carcinogenesis. Current risk factor models do not include direct measures of cycle characteristics although the effects of possible surrogates of cycle activity such as age at menarche and menopause, parity, and nursing time have been investigated. Future risk models should also include menstrual cycle length, regularity, number of cycles before first full-term pregnancy, and life-time number of cycles. New risk factor models for pre- and postmenopausal breast cancer are proposed here. Furthermore, there is a need for more long-term, prospective studies investigating menstrual cycle characteristics as data currently available are primarily retrospective and collected at one time-point only.

## Background

In the 1990s, our research group pioneered studies on menstrual cycle length, menstrual regularity, and the number of menstrual cycles as risk factors for breast cancer (1, 2). Women who developed breast cancer were more likely to have short, regular cycles, and had more cycles before the first full-term pregnancy than healthy women and those with benign breast disease. As the luteal phase is fixed in time, only the follicular phase may vary, thus exposing women with shorter, and more numerous cycles to higher amounts of progesterone during the luteal phase (3). We and others have also shown a greater number of dividing epithelial cells in the luteal phase than in the follicular phase (4–6). Cell division is generally considered a prerequisite for carcinogenesis and women with short and numerous cycles may therefore have a higher risk of developing cancer as a result of increased cell proliferation. Although progesterone protects against endometrial cancer, it appears to have a different effect in increasing breast cancer risk (7). This was confirmed by recent findings investigating breast cancer type 1 susceptibility protein (BRCA1) carcinogenesis, the roles of progesterone and receptor activator of nuclear factor kappa-B ligand (RANKL), and the therapeutic potential of anti-progestins (8, 9).

Furthermore, several studies regarding the risk of exogenous hormones and breast cancer revealed that the combination of progestins and estrogen increased the risk of breast cancer compared with the effects of estrogen alone (10–13). We also showed that shorter menstrual cycles were associated with the cytochrome P450 17 (CYP17) genotype (14).

A list of studies concerning the menstrual cycle is presented in Table 1 (15–25). These studies indicate that a high number of cycles before the first full-term pregnancy and high life-time menstrual activity (LMA) increased breast cancer risk. Furthermore, a short time interval between menarche and the establishment of regular cycles is another risk factor. In contrast, no relationship was observed between the length of menstrual bleeding and breast cancer (26). Of the studies listed in Table 1 two (16, 20) included only Asian women and one (24) only American African women.

**Table 1:** Studies of different menstrual cycle characteristics and breast cancer risk.

**Study / year**	**Type of study**		**Main effect**				
		**Short cycles**	**Long cycles**	**NC < AFFP**	**LMA**	**Regularity**	**Comment**
Olsson et al. (1)	Case-control	+	–	na	na	+	
Olsson et al. (2)	Case-control	+	–	+	na	+	
Bernstein et al. (15)	Case-control	na	na	na	(+)	na	
Yuan et al. (16)	Case-control	+	0	na	na	0	
Rautalahti et al. (17)	Case-control	na	na	na	+	na	
Whelan et al. (18)	Cohort	+	+	na	+	na	Also effect of long cycles
den Tonkelaar et al. (19)	Case-control	na	na	na	+	+	
Chie et al. (20)	Case-control	na	na	+	na	na	
Titus-Ernstoff et al. (21)	Case-control	0	0	Increased risk if short time between puberty to menstrual regularity Reduced risk if early surgical menopause			
Garland et al. (22)	Cohort	–	–	na	+	+	
Clavel-Chapelon and E3N Group (23)	Case-control	na	na	+	+	na	
Beiler et al. (24)	Case-control	+	–	na	na	na	
Chaves-MacGregor et al. (25)	Case-control	na	na	+	+	+	

*Short cycles, average cycle in general <26 days; Long cycles, average cycle in general longer than 33 daysl NC < AFFP, number of menstrual cycles before first full term pregnancy; LMA, life time menstrual activity or number of life time cycles; Regularity, regular menstrual cycles; na, not assessed; +, increased risk; –, decreased risk; 0, neutral findings*.

LMA is calculated for natural cycles using the following variables: age at menopause and menarche, average cycle length, number of pregnancies, and duration of nursing excluding periods of exogenous hormone use. There are however a number of relevant caveats: first, cycle length may vary during reproductive life and studies thus consider the average cycle length. In retrospective studies, there may be a recall bias for cycle length. Furthermore, there are discrepancies regarding the number of cycles counted during exogenous hormonal treatment (27, 28). In addition, there are few high-quality, long-term (life-time) prospective studies investigating cycle length. In this context and in support of the importance of LMA, it is notable that early menopause or castration protect against breast cancer. Other factors such as extreme physical activity and starvation reduce cyclic activity and thus breast cancer risk (29). Finally, the consistency in results regarding cycle length, the number of cycles before the first full-term pregnancy, and LMA indicate that the crude retrospective assessment of menstrual cycles has an important bearing on investigating breast cancer risk.

Benign breast disease is characterized by irregular menstrual cycles and is more common at the end of reproductive life (1). Irregular cycles cause cystic disease in the breasts and ovaries and women with cystic ovarian disease therefore have a lower incidence of breast cancer (30).

We have postulated that women whose breast size is maintained or increased after hormonal exposure may have a higher risk of cancer than those whose breast size decreases upon such exposure (31). However, this hypothesis requires further investigation of the menstrual cycle. Possible assessment of breast density or magnetic resonance imaging (MRI) images without contrast assessing fibroglandular density may be helpful (32).

Finally, the effects of oral contraceptive (OC) use should be investigated. For example, it is unclear whether lengthening menstrual cycles artificially via administration of OCs in women with naturally short cycles decreases cancer risk. Conversely, it is also unclear whether cancer risk increases in women whose naturally long cycles are artificially shortened by the use of OCs.

A number of risk factors have been identified for breast cancer such as age at menarche, age at first full term pregnancy, parity, age at menopause, obesity (postmenopausal risk), number of menstrual cycles, weight gain, hormone replacement therapy, early oral contraceptive use, breast size, preecclampsia, birth weight, nursing, height, breast density, physical activity, night shift work, radiation exposure, tobacco use, alcohol use, family history, mutation carrier of a predisposing gene. Some of the above factors are still under investigation with partly diverging findings such as for tobacco use, breast size and night shift work and others like preecclampsia and high physical activity are protective. Some factors like radiation exposure, reproductive and genetic factors are more important premenopausally, while obesity is more important for older women.

Development of better methods to describe the menstrual cycle more exact is needed. One method is of course to use a calendar recording the start of each menstruation, another way is to record basal body temperature daily, women in the luteal phase have a higher body temperature, or study the cervical mucus. However, it can be difficult to pinpoint ovulation using these methods, especially if your menstrual cycles are irregular. Research in fertility medicine especially in women with irregular menstruations is mainly driven to better time ovulation through ovulation prediction kits either using urine (measuring LH) or saliva (studying ferning patterns in relation to estrogen increase). Again these latter methods are too cumbersome and expensive to be used in large epidemiological risk factor studies and explain their absence in literature.

## Conclusion and Proposal

The characteristics and number of menstrual cycles before the first full-term pregnancy, LMA, and menstrual regularity require further investigation as part of epidemiological studies of breast cancer, as other risk factors such as age at menarche and menopause, parity, and nursing are only surrogates for cyclic hormonal exposure. Menstrual cycle characteristics should be included in risk factor models of breast cancer. Current models such as Gail, Tyrer-Cusick, Rosner Colditz BCRAT, BCPRO, and BOADICEA only include family history, germline mutation status, breast density, polygenic risk scores, and surrogates of cycle activity such as age at menarche, age at first full-term pregnancy (AFFP), parity, nursing, and age at menopause (33–39). The BOADICEA and Tyrer-Cusick models appear to be the most informative (39). Parity and AFFP may exert independent effects on differentiation of the breast epithelium, and are indirectly related to menstrual cycle activity. However, cyclic hormonal stimulation of the breast tissue, which is probably the most important hormonal factor contributing to breast cancer, is not directly investigated in such models. Proposed revised risk factor models for pre- and postmenopausal breast cancer are listed in Table 2. Only surrogates such as age at menarche, AFFP, parity, and nursing have been included in previous studies. Prospective life-time studies on menstrual cycle activity are encouraged, as current studies primarily include retrospective data collected at one time-point and often use average measures of menstrual factors. Studies covering longer time periods should include other factors of importance for the menstrual cycle such as physical activity, obesity, psychological stress, and intercurrent diseases such as osteoporosis (29).

**Table 2:** Revised risk factor models for breast cancer taking the menstrual cycle into account.

**Classic**	**Revised premenopausal**	**Revised postmenopausal**
Family history Germline mutations Polygenic risk score Breast density Age at menarche AFFP Age at menopause HRT use	Family history Germline mutations Polygenic risk score Breast density NC < AFFP (parity, AFFP) OC use Regular cycles Physical activity	Family history Germline mutations Polygenic risk score (Breast density) LMA (parity, AFFP) HRT use Regular cycles Weight/weight gain

*NC < AFFP, number of menstrual cycles before first full term pregnancy; LMA, life time menstrual activity or number of life time cycles*.

*AM, age at menarche; AAFP, age at first full term pregnancy; OC use, oral contraceptive use; HRT use, hormone replacement therapy use*.

## Author Contributions

All authors listed have made a substantial, direct and intellectual contribution to the work, and approved it for publication.

### Conflict of Interest

The authors declare that the research was conducted in the absence of any commercial or financial relationships that could be construed as a potential conflict of interest.
